# Performance prediction of sintered NdFeB magnet using multi-head attention regression models

**DOI:** 10.1038/s41598-024-79435-7

**Published:** 2024-11-21

**Authors:** Qichao Liang, Qiang Ma, Hao Wu, Rongshun Lai, Yangyang Zhang, Ping Liu, Tao Qi

**Affiliations:** 1 Department of Rare Earth, Jiangxi University of Science and Technology, Ganzhou, 341000 China; 2 Department of Physics, Ganjiang Innovation Academy, Chinese Academy of Sciences, Ganzhou, 341119 China; 3 Department of Technology, Jiangxi Guanying Intelligent Technology Co.Ltd., Ganzhou, 341000 China

**Keywords:** Sintered NdFeB, Machine learning, Deep learning, Multi-head self-attention mechanism, XGBoost, Magnetic properties and materials, Statistics

## Abstract

The preparation of sintered NdFeB magnets is complex, time-consuming, and costly. Data-driven machine learning methods can enhance the efficiency of material synthesis and performance optimization. Traditional machine learning models based on mathematical and statistical principles are effective for structured data and offer high interpretability. However, as the scale and dimensionality of the data increase, the computational complexity of models rises dramatically, making hyperparameter tuning more challenging. By contrast, neural network models possess strong nonlinear modeling capabilities for handling large-scale data, but their decision-making and inferential processes remain opaque. To enhance interpretability of neural network, we collected 1,200 high-quality experimental data points and developed a multi-head attention regression model by integrating an attention mechanism into the neural network. The model enables parallel data processing, accelerates both training and inference speed, and reduces reliance on feature engineering and hyperparameter tuning. The coefficients of determination for remanence and coercivity are 0.97 and 0.84, respectively. This study offers new insights into machine learning-based modeling of structure-property relationships in materials and has potential to advance the research of multimodal NdFeB magnet models.

## Introduction

With the development of modern technology and the continuous expansion of the electric vehicle market, the demand for high-performance sintered NdFeB permanent magnet materials is increasing. Machine learning methods can accelerate the optimization of material composition, processes, and performance. Traditional machine learning models, based on mathematical and statistical principles, such as linear regression (LR) and its variants, support vector machines (SVM)^1^, random forests (RF)^2,3^, and gradient boosting trees (GBT)^4^, are widely used in materials science due to their stability and reliability. Currently, research on sintered Nd-Fe-B materials primarily relies on these traditional models due to limitations in data scale. In 2021, Zhang^5^ collected 25 sets of magnet experimental data containing five elements and remanence, using multiple linear regression(MLP) and SVM models to predict the remanence. In 2023, Kini^6^ expanded on this work by introducing RF and voting regression models, using 189 data points with 33 elements to predict mass density. Due to the small size of both datasets and the strong linear relationship between the feature and target variables, the accuracy of the MLP model surpassed that of other regression models.

Also in 2023, Qiao^7^ extracted 262 data points from the literature, which included 20 elements, remanence, coercivity, and sintering processes, increasing both the scale and complexity of the data. The results demonstrated that the GBT model outperformed SVM and RF models, achieving coefficients of determination of 0.957 and 0.923 for predicting remanence and coercivity, respectively. The SHAP^8^ method was employed to illustrate the nonlinear relationship between feature variables and coercivity. The GBT model is an ensemble learning approach^9^ based on decision trees^10^ that enhances the model’s generalization ability by integrating multiple classification and regression trees^11^. Drawing on the principles of ensemble learning, Amit, in 2024, collected 198 data points containing 28 elements and saturation magnetization and introduced a non-uniform weighted voting regression model. This model aggregates the outputs of four different models: linear Huber regression, RF, adaptive boosting decision trees, and multilayer perceptrons, using weighted voting to predict saturation magnetization. The findings indicate that the ensemble learning model improves prediction accuracy. At present, traditional machine learning models, represented by extreme gradient boosting (XGBoost)^12^ and LightGBM^13^, are extensively applied to classification and regression tasks.

As the scale and variety of data increase, the computational complexity of traditional machine learning models in feature engineering, model training, and hyperparameter tuning rises significantly. However, the successful application of deep learning^14^ in computer vision, speech recognition, and natural language processing demonstrates that neural network-based deep learning models can capture nonlinear relationships among features in large-scale data. These models can also handle multimodal data, including structured data such as composition, process, and performance, as well as unstructured data, like microscopic images^15^ of magnets and crystal structures^16^. Neural networks are composed of multiple layers of nodes, which typically results in lower interpretability compared to traditional machine learning models. To improve interpretability, we introduce attention mechanism^17,18^ into standard deep neural network models, constructing a multi-head attention regression model^19^. This model transforms the relationships between feature variables into weight parameters, helping to capture and display long-range dependencies among features. In this study, we collect over 1,200 high-quality data points on sintered NdFeB compositions, processes, and performances, with 32 features. We apply extreme gradient boosting(XGB), standard deep neural networks regression (DNNR), and multi-head attention regression(MHAR) models to predict four target variables: remanence, coercivity, maximum magnetic energy product, and squareness. By analyzing and comparing traditional machine learning models and deep neural network models in terms of data analysis, model performance, hyperparameter tuning, and interpretability, this research offers new machine learning approaches for studying sintered NdFeB materials.

## Results

### Data analysis

**Fig. 1:**
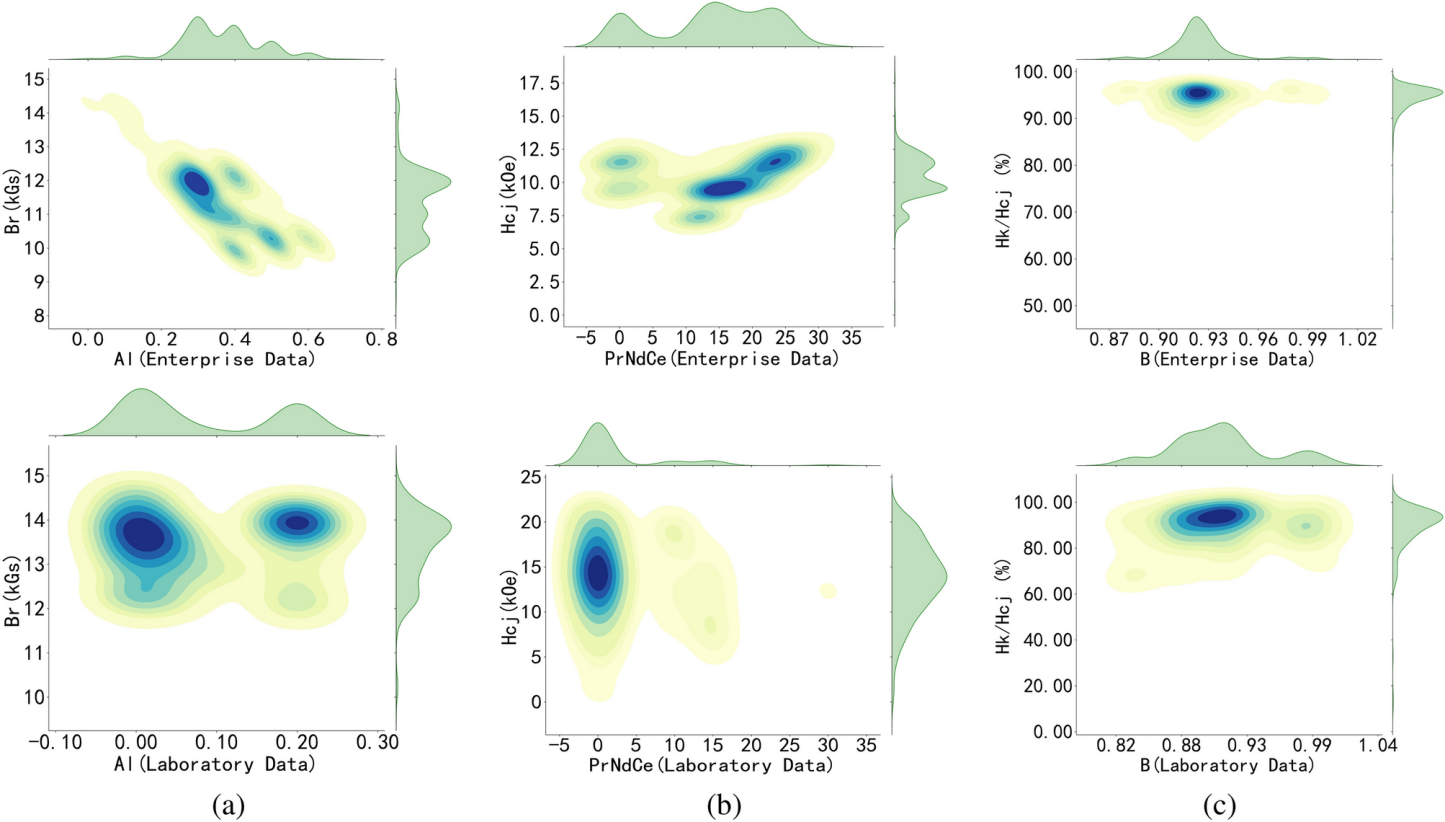
The joint distribution maps of features and target variables. (**a**) Distribution of Al elements with remanence in laboratory and enterprise data; (**b**) PrNdCe elements with coercivity; (**c**) B elements with squareness..

Feature engineering is a key step in traditional machine learning, where features are selected, constructed, and transformed by observing and analyzing data distributions to enhance model performance. Figure 1 shows the joint distribution of Al, B, and PrNdCe with remanence, coercivity, and squareness in enterprise and laboratory data. The figure reveals significant differences in data distribution between the enterprise and laboratory datasets. In the enterprise data, a clear negative correlation between the Al element and remanence is observed, while PrNdCe shows a positive correlation with coercivity.

The distribution chart also indicates that most magnets produced in laboratories did not contain Al, and only a small number of magnets had 0.2% Al added, with minimal impact on remanence. Due to the limited number of magnet samples containing PrNdCe, its effect on coercivity could not be determined. Both the enterprise and laboratory added approximately 0.9% of the B element, however, the squareness of the magnets produced by the company is superior to that of the laboratory samples, indicating better consistency in the enterprise data.

The primary objective of enterprise is to reduce costs and increase efficiency, which necessitates mass production. As a result, enterprise data is characterized by large volumes, significant variation in magnet composition, high consistency in magnet performance, but a single sintering process. In contrast, laboratory data is typically generated to address specific scientific problems, resulting in smaller datasets for each experiment, but with considerable variation in composition, processes, and performance across experiments. Integrating enterprise data with laboratory data can offset their respective limitations and enhance data diversity. The integrated data shows that the distribution of remanence and maximum magnetic energy product is highly consistent, indicating a linear relationship between these variables. The squareness of the magnets generally falls between 80% and 90%, with no significant correlation to other features.

### Model performance analysis

We uses three metrics to evaluate model performance: Root Mean Squared Error^20^ (RMSE), Mean Absolute Error^21^ (MAE), and the coefficient of determination^22^ ($R^2$). RMSE represents the deviation between predicted values and actual values, while MAE indicates the average degree of deviation of predicted values from actual values. The smaller these two values are, the better the model’s predictive performance. $R^2$ reflects the goodness of fit of the data, with values closer to 1 indicating a better fit.

The performance metrics of three models—XGB, DNNR, and MHAR—on laboratory, enterprise, and integrated datasets are presented in Table 1. Each dataset is evaluated using three performance metrics: RMSE, MAE, and $R^2$. The models are assessed across four material properties: remanence, coercivity, maximum energy product, and squareness. The results indicate that prediction accuracy improves for all three models as the data scale increases, highlighting the positive influence of both data volume and diversity on model performance. However, the multi-head attention regression model underperforms compared to XGBoost, likely due to overfitting caused by the smaller dataset during complex model training. Although MHAR and DNNR exhibit similar predictive accuracy across datasets, MHAR offers higher computational efficiency and better interpretability. Additionally, all three models exhibit significant errors in predicting squareness, primarily due to the absence of feature variables directly related to squareness. Despite these issues, all three models produced satisfactory prediction results overall.

**Table 1:** Performance comparison of XGB, DNNR, and MHAR models across different datasets.

Model	Property	Laboratory Data			Enterprise Data			Integrated Data		
		RMSE	MAE	$R^2$	RMSE	MAE	$R^2$	RMSE	MAE	$R^2$
XGB	$B_{\text {r}} \text {(kGs)}$	0.31	0.16	0.79	0.18	0.11	0.97	0.16	0.11	0.98
	$H_{\text {cj}} \text {(kOe)}$	1.29	0.88	0.88	0.57	0.36	0.89	0.74	0.41	0.93
	$(BH)_{\text {max}} \text {(MGOe)}$	2.49	1.35	0.80	0.96	0.64	0.97	1.39	0.76	0.96
	$H_{\text {k}}/ H_{\text {cj}} {(\%)}$	9.91	5.30	0.49	4.15	1.84	0.02	3.83	1.92	0.51
DNNR	$B_{\text {r}} \text {(kGs)}$	0.61	0.49	0.17	0.19	0.14	0.96	0.22	0.16	0.96
	$H_{\text {cj}} \text {(kOe)}$	2.12	1.75	0.67	0.71	0.47	0.83	1.16	0.75	0.83
	$(BH)_{\text {max}} \text {(MGOe)}$	4.39	2.61	0.38	1.14	0.83	0.96	1.99	1.11	0.93
	$H_{\text {k}}/ H_{\text {cj}} {(\%)}$	11.99	7.39	0.26	4.22	2.06	-0.01	4.84	2.67	0.22
MHAR	$B_{\text {r}} \text {(kGs)}$	0.35	0.22	0.72	0.20	0.14	0.96	0.22	0.16	0.97
	$H_{\text {cj}} \text {(kOe)}$	1.80	1.38	0.77	0.72	0.48	0.83	1.15	0.72	0.84
	$(BH)_{\text {max}} \text {(MGOe)}$	4.65	2.74	0.31	1.15	0.81	0.96	2.09	1.14	0.92
	$H_{\text {k}}/ H_{\text {cj}} {(\%)}$	12.16	7.32	0.23	4.32	2.08	-0.06	4.90	2.64	0.20

**Fig. 2:**
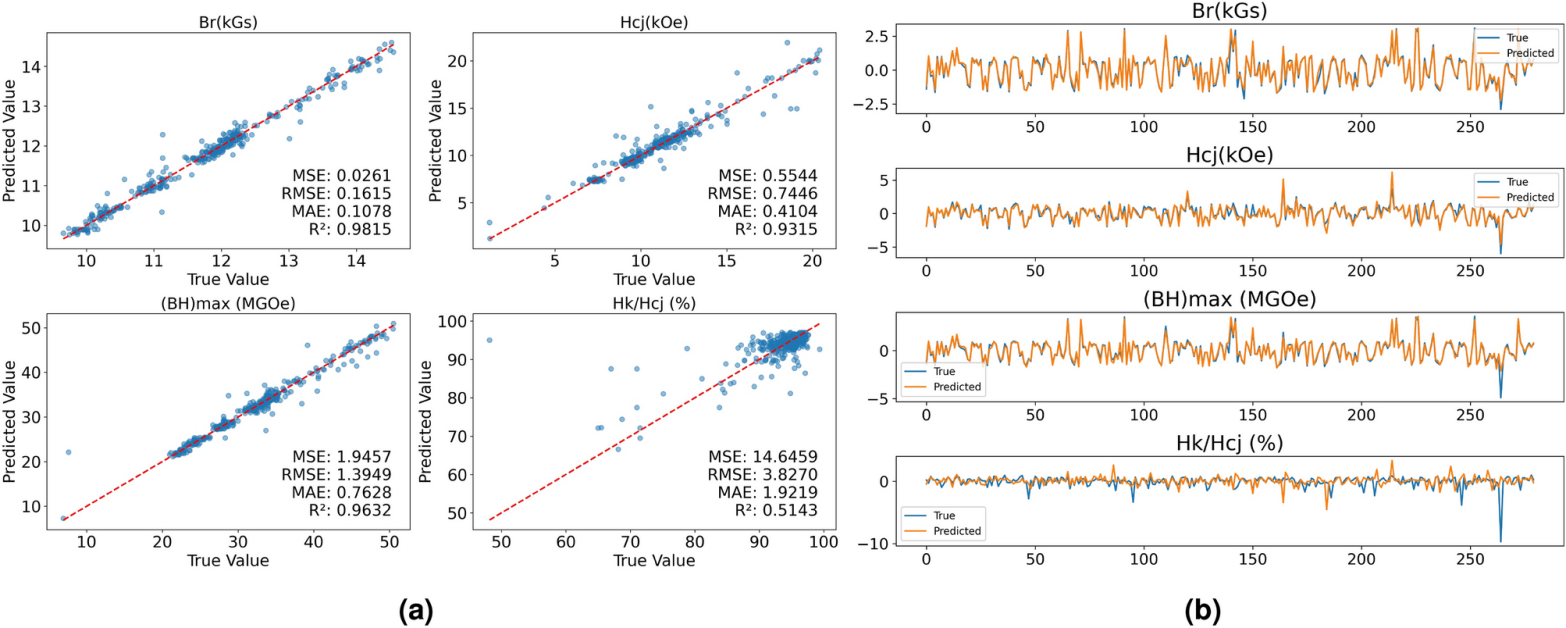
(**a**) Represents the performance of the XGBoost model on testing data, and (**b**) shows the performance of the multi-head attention regression model on testing data.

To more intuitively demonstrate the predictive performance of the models, Fig. 2 shows the prediction results of the XGBoost model and the multi-head attention regression model on the test set of the integrated data. In Fig. 2a, The blue dots represent the sample data in the test set and the red line represents where the predicted values equal the actual values. The closer the sample points are to the red line, the better the model’s prediction performance. In Fig. 2b, the horizontal axis represents the number of test data samples, and the closer the yellow and blue lines are to each other, the better the model’s predictive accuracy. Both models achieve a fitting degree of over 95% for remanence and magnetic energy product, while the prediction error for squareness is relatively large, indicating a poor fit. As shown in Fig. 2b, the curves representing the true and predicted values of squareness show minimal overlap, suggesting an absence of feature values in the training data that significantly influence squareness.

### Hyperparameter tuning

Hyperparameters are key factors that affect both the learning process and the final performance of a model. The complexity, learning capacity, and generalization ability of the model can be optimized by hyperparameters, achieving better prediction accuracy or performance on specific tasks.

The XGBoost model has a total of 40 adjustable parameters. Nine parameters that have the most significant impact on model performance were selected for optimization. After the models trained using default parameters, it can output the hyperparameter values that need to be adjusted. The regions around these values are selected as the search space, and bayesian optimization is used to find the best combination of hyperparameters within this space. Optimizing all hyperparameters simultaneously consumes more computational resources and time than training the model itself.

**Fig. 3:**
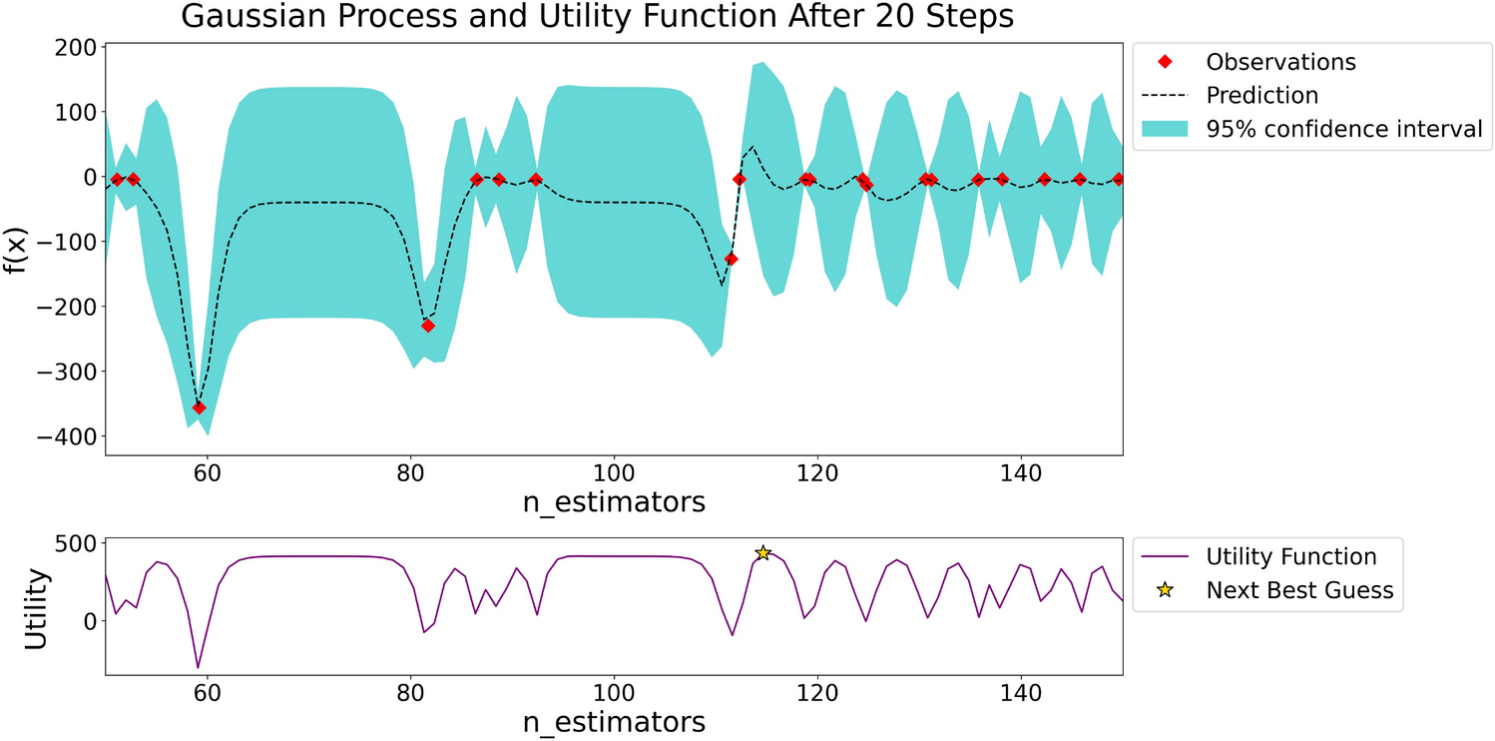
The gaussian process regression and utility function results after the 20th iteration.

To further improve optimization efficiency, specific tuning strategies are recommended. Initially, adjust parameters related to the complexity of the tree model, such as the number of trees, maximum depth, and minimum leaf node weight. Subsequently, address parameters that prevent model overfitting and those related to data sampling strategies, followed by the adjustment of the model’s learning rate. Figure 3 illustrates the gaussian process regression and utility function results after the 20th iteration of adjusting the number of trees hyperparameter.

Bayesian optimization^23^ optimizes the hyperparameters of a model through gaussian process^24^ regression and utility functions^25^. The upper part of Fig. 3 represents the gaussian process regression, where the red dots indicate the results of the model calculations for the target function under different hyperparameter combinations. The dashed line represents the predictive curve of the gaussian process regression for unknown parameter combinations of the target function. The blue area is called the 95% confidence interval, which indicates that the values of the target function calculated by the model under different combinations of hyperparameters have a 95% probability of falling within this range. The confidence interval quantifies the uncertainty of model prediction, as the number of observed points increases, the confidence interval becomes narrower, making the parameter estimation more accurate.

The lower part of Fig. 3 represents the utility function, where the pentagon denote the next observation point to be calculated. The parameter combination of this point is the optimal parameter combination found by the Bayesian optimization algorithm in the search space. As the number of iterations increases, the Bayesian optimization algorithm gradually converges to the optimal parameter point, and ultimately finds the global optimum. The XGB model has numerous parameters, and within the given search space, bayesian optimization still requires lengthy computation time to achieve convergence.

Deep learning models are structurally more complex than traditional machine learning models. In this experiment, we employed ReLU activation function, MAE loss function, and Adam optimizer to train the deep neural network. To prevent overfitting during the training process, parameters such as the number of layers, the number of neurons, and batch size are set within a narrow adjustment range, while the regularization coefficient and learning rate are chosen based on empirical values. In contrast, the hyperparameters of the multi-head attention regression model only add one parameter, which is the number of heads. Given the limitations of data size, the number of heads should be controlled appropriately, with a recommendation to set it as an integer within 4.

### Interpretability of models

Model Interpretability refers to the ability to explain how it makes predictions or decisions. In this paper, the XGBoost model is a tree-based ensemble learning model, where the tree structure can intuitively represent the decision-making process.

**Fig. 4:**
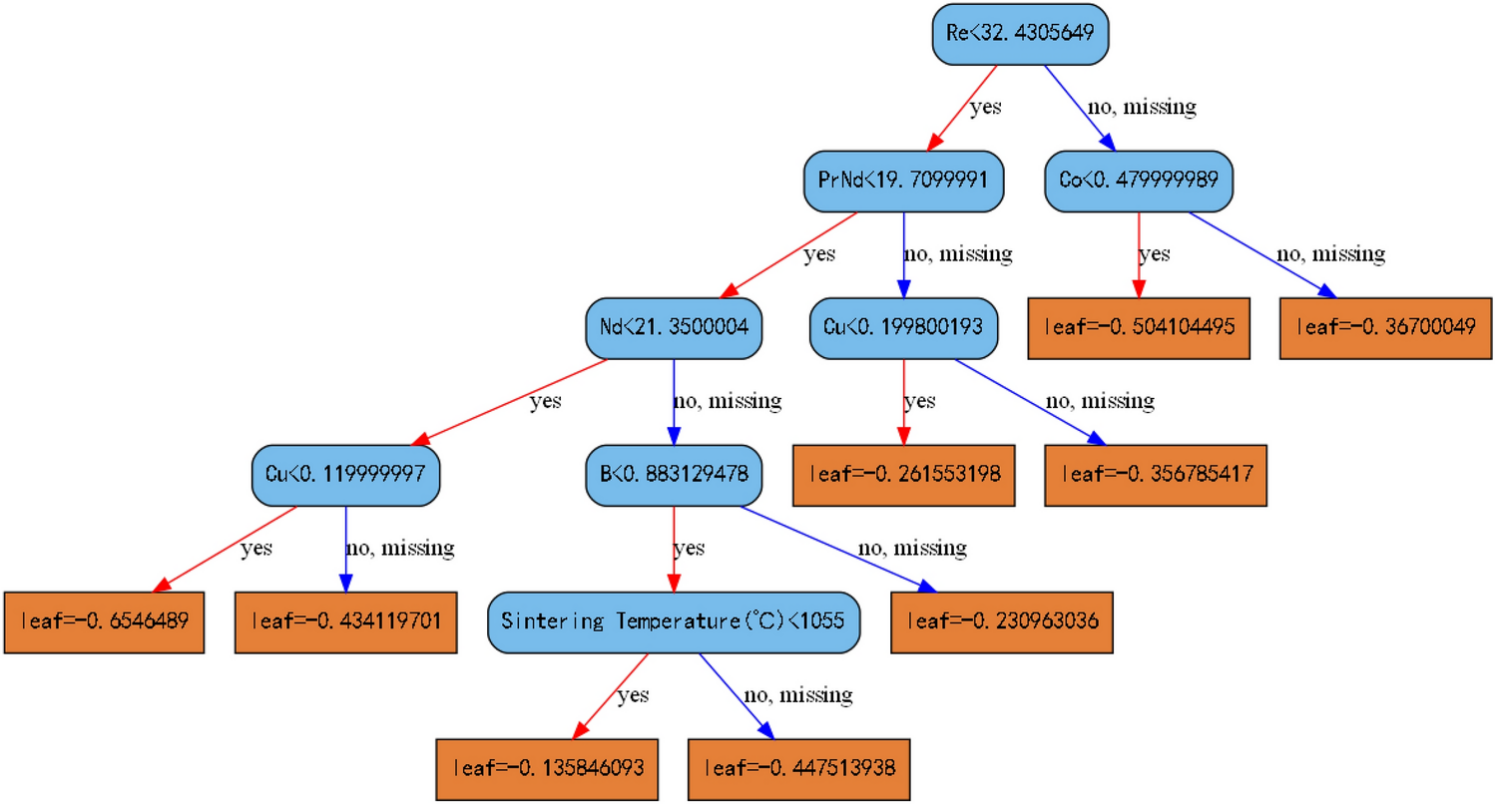
The structure diagram of the 32rd decision tree.

Figure 4 depicts the structure of the 32rd decision tree, with a maximum depth of 5. All sample data were partitioned into 9 leaf nodes according to the rules of the decision tree, and the output value of each leaf node is the weighted average of the residuals of all samples at that point. Adding this to the prediction value from the previous tree will yield a new prediction value. By continually building new trees to correct the residuals of the previous tree, the prediction value is gradually optimized. Therefore, the decision-making process of traditional machine learning models based on mathematical principles is highly transparent and offers strong interpretability.

SHapley Additive exPlanations^8^ (SHAP) is a model interpretation method based on additive decomposition, which can evaluates whether a feature has a positive or negative impact on the prediction result by calculating the average marginal contribution of each feature in all possible feature subsets, known as SHAP values.

**Fig. 5:**
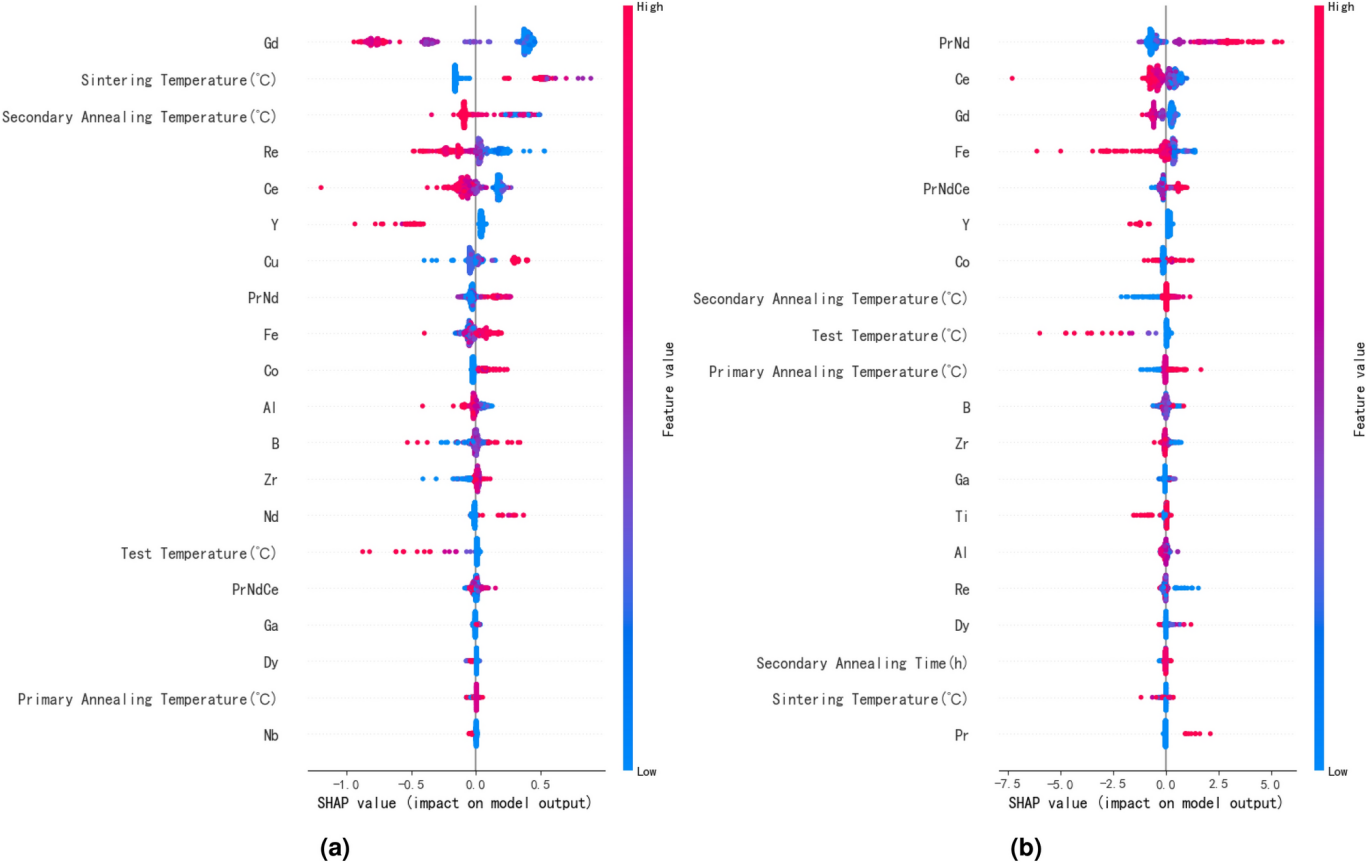
Figure show the global view of feature importance, (**a**) and (**b**) represent the importance of feature variables on remanence and coercivity respectively.

Since the influence of feature data on remanence and maximum energy product exhibits a similar trend and has no significant relationship with squareness, Fig. 5 only illustrates the impact of various feature variables on remanence and coercivity in an XGBoost model. Each point in the figure represents a sample, with redder points indicating larger feature values. It can be observed from Fig. 5a that the feature values of elements such as Gd, Ce, Al, and Y increase in the direction of decreasing SHAP values, indicating that an increase in the content of these elements have an inhibitory effect on remanence. Similarly, Fig. 5b shows that PrNd, Co, and tempering processes can increase the coercivity of the magnet. By combining Figures a and b, it can be observed that secondary annealing may reduce some remanence while improving coercivity, indicating that this process is conducive to promoting phase transformation. Based on SHAP values, components and processes that enhance magnet performance can be quickly identified, accelerating the development of high-performance magnets.

**Fig. 6:**
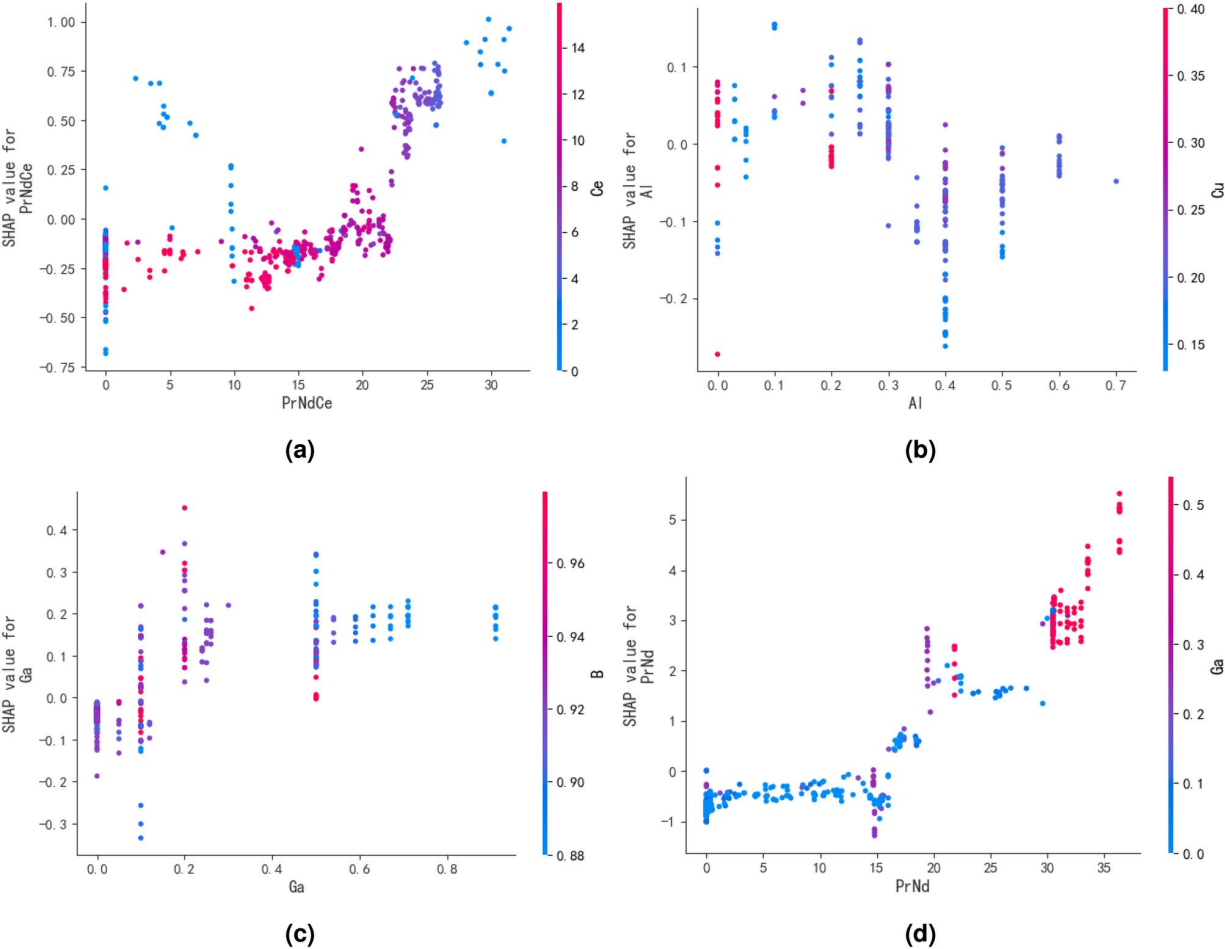
Figure show the impact of interactions between any two features on the target variable, (**a**–**d**) represent the synergistic effects between PrNdCe and Ce, Al and Cu, Ga and B and PrNde and Ga, respectively.

SHAP can also illustrates the impact of interactions between any two features on the target variable,which aids in studying the synergistic effects between different elements. Figure 6 shows the influence of interactions between two features on the coercivity of magnets. Figure 6a indicates that the addition of a small amount of Ce element to PrNdCe within the range of 20–25% does not decrease coercivity, which contributes to reducing the cost of magnet preparation. Figure 6b demonstrates that the addition of Cu has a positive effect on coercivity enhancement when the Al content exceeds 0.4%. Additionally, adding 0.1% each of Al and Cu results in the maximum enhancement of the magnet’s coercivity. Figure 6c reveals that with the increase of Ga element, the gradual decrease in B content has a positive effect on coercivity enhancement, validating that the high Ga low B system can enhance the coercivity of magnets. Figure 6d illustrates that as the rare earth element PrNd increases, the promoting effect of Ga on the coercivity of the magnet becomes more significant, indirectly confirming that the $Pr_6Fe_{13}Ga$ phase, which is beneficial for enhancing the performance of the magnet, needs to be formed in an environment with excess rare earth elements.

**Fig. 7:**
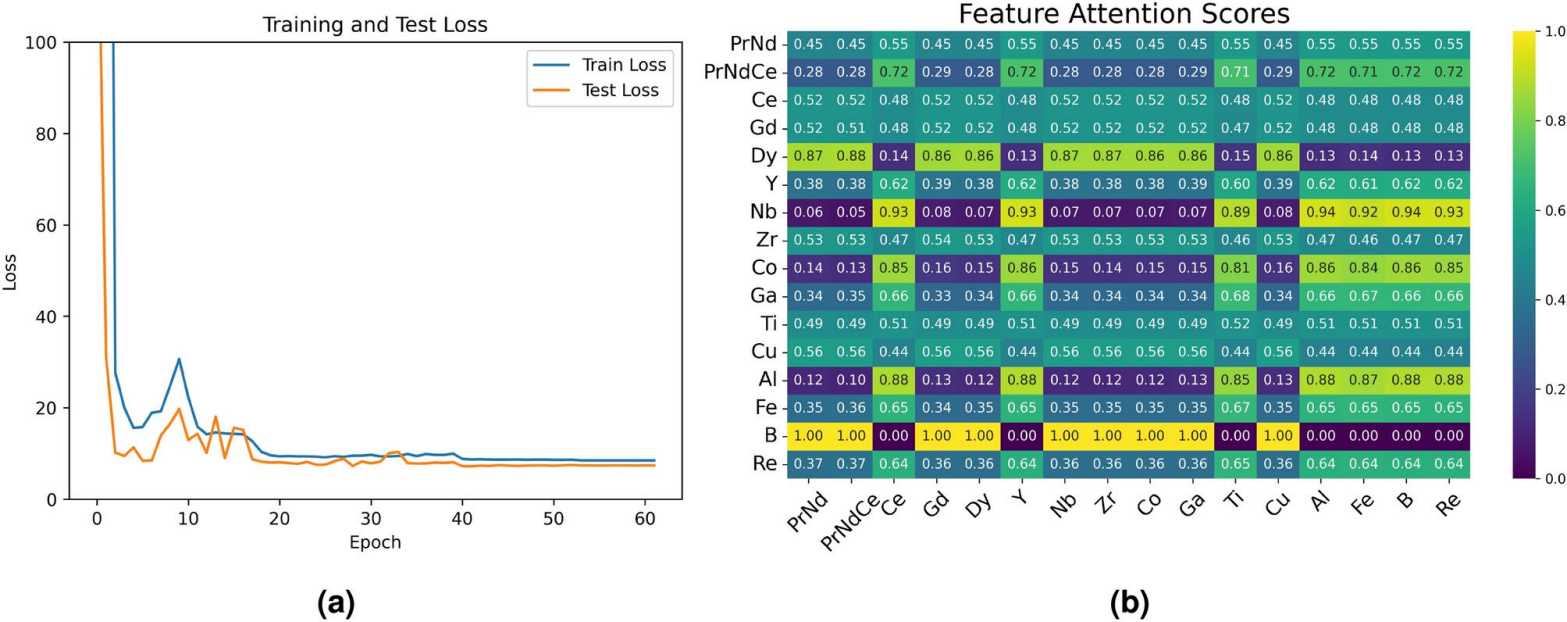
(**a**) Represent the changes in the loss functions of the train and test sets during the training process and (**b**) shows the attention scores between features.

Neural network models capture the weight relationships between features through neurons in hidden layers. The initial parameters carried by these neurons are randomly generated and do not represent any physical significance, making it difficult to explain how neurons influence model training. The importance of features calculated through the attention mechanism can be used for the weight parameters of neurons, improving the model’s interpretability. Figure 7 demonstrates the interpretability of the attention regression model. Figure 7a shows the change in loss for the training and test sets during the training process. It can be seen that the model stops training after 60 iterations, indicating that the early stopping mechanism effectively prevents overfitting. Figure 7b shows the attention scores between components. The attention score map reveals that the average attention scores between the added trace elements and other feature variables are relatively high, suggesting that incorporating trace elements such as Zr, Co, Ga, and Ti enhances magnet performance. In contrast, variables like B exhibit strong correlations with specific variables, indicating that the microstructure of magnets can be optimized by adjusting these highly correlated factors. Attention scores have improved the interpretability of neural networks to some extent, and providing an important approach for parallel processing of large-scale data.

## Discussion

This study presents a comparative analysis of traditional symbolic-based machine learning models and deep neural network models in terms of data analysis, model performance, hyperparameter tuning, and model interpretability. A multi-head attention regression model is proposed based on a standard deep neural network regression model. This model exhibits higher complexity than traditional machine learning models, making it susceptible to overfitting on small datasets, which leads to lower predictive accuracy compared to the XGBoost model. However, its complexity does not significantly increase with larger and more diverse datasets. In contrast, traditional machine learning models require deeper trees or larger search spaces when processing high-dimensional and large-scale data, increasing the time and complexity of model training and hyperparameter tuning, and potentially resulting in failure to converge. While the interpretability of the multi-head attention regression model is inferior to that of traditional machine learning models, it still improves the interpretability of neural network models to some extent. Enhancing the interpretability of deep neural network models remains a critical focus in machine learning research.

Two datasets from the laboratory and enterprise were collected in the experiment. The laboratory data exhibit a more favorable distribution compare to the enterprise data, which is overly uniform due to its process-related nature. However, the laboratory dataset is only one-fourth the size of the enterprise dataset. By combining both datasets for training, the three models showed higher accuracy on the merged data than on either dataset individually, indicating that increased data scale and diversity enhance model performance. Notably, all the models show a large prediction error for the squareness, suggesting that it is not directly related to the composition and process of the magnets. The composition of the grain boundary phases may serve as valuable features to enhance the accuracy of squareness predictions. When analyzing the contribution of components and processes to magnet performance with SHAP values, it was found that the SHAP value of the heavy rare earth element Dy, known for significantly enhancing magnet coercivity, is relatively low. This is attributed to the limited number of magnet samples containing Dy in the dataset, which prevents it from capturing the full effect of heavy rare earth elements on coercivity.Feature combination analysis revealed that trace elements Al and Cu in magnets exist in optimal proportions and that materials with a high Ga and low B system promote coercivity enhancement. These hidden relationships can inform the development and theoretical research of high-performance NdFeB materials. To further improve the model’s predictive accuracy, expanding the dataset by incorporating more data from the literature to increase the scale and diversity of features is recommended.

The neural network-based multi-head attention regression model is not only suitable for processing large-scale structured data but also possesses strong scalability. By introducing different functional modules to improve the model, such as adding convolutional neural networks for image data, attention networks for sequence data like X-ray diffraction, and graph neural networks for crystal structure data, the model can be adjusted to a large architecture with encoders and decoders. This allows for modeling any type of data^26^ related to sintered NdFeB materials. As the data expands to include more types of permanent magnet materials, further insights can be gained regarding the differences and connections between NdFeB materials and other permanent magnets. If a large language model is integrated into the framework, it can provide inferences based on the output results of each module, offering appropriate suggestions for specific scientific problems. A multimodal intelligent large model based on deep learning may become an important direction for machine learning research in the field of materials.

## Conclusion

This study collected three datasets on sintered NdFeB materials, with the largest dataset comprising over 1,200 entries from laboratory and enterprise. Machine learning methods were employed to investigate the relationships between the composition, process, and performance of sintered NdFeB magnets, the following conclusions were drawn:

1. The standard neural network regression model exhibits strong nonlinear modeling capabilities, capable of handling data of varying scale and type. Building on this, we proposed a multi-head attention regression model. The introduction of the attention mechanism enhances the interpretability of the neural network, while the multi-head structure facilitates parallel data processing, thereby improving computational efficiency.
2. Although the interpretability of the multi-head attention regression model is less than that of traditional machine learning models based on mathematical symbols, it can automatically identify relationships between feature values via the attention mechanism, reducing the reliance on feature engineering. Even without complex hyperparameter tuning, the model achieved an average fit of over 0.9 for the three key performance indicators of the magnets.
3. As the dataset size increases, all three regression models show a decrease in errors on the test set and an improvement in fit, indicating that the scale and diversity of the data enhance the training accuracy of the machine learning models.
4. Data mining revealed that when the addition of Al exceeds 0.4% or when Gd and Y are added, the remanence and coercivity of the magnets are suppressed. However, when the mixed rare earth content of PrNdCe in the magnet reaches the 20%-25% range, adding an appropriate amount of Ce still helps to improve the magnet’s coercivity. Additionally, the study of the synergistic effects of B and Ga confirmed that a high Ga and low B material system contributes to enhanced magnet coercivity.
5. The multi-head attention regression model exhibits strong scalability. When integrated with convolutional neural networks, it can simultaneously process structured macroscopic performance data and unstructured microscopic image data, establishing a connection between the two, thereby advancing research on multimodal permanent magnet material models.

## Methods

### Data collection

1225 sets of sintered NdFeB magnet data have been collected, including 977 sets of production data from enterprises and 248 sets of data from laboratory-prepared magnets, which is currently the largest dataset in this field. The data consists of three parts: composition, process, and performance of the magnet. The magnet composition includes 10 rare earth elements, 7 trace elements, as well as Fe and B elements. Process data includes the temperature and time of one sintering and two annealing processes. Performance data includes remanence, coercivity, maximum magnetic energy product, squareness, particle size, density, and oxygen content. Among these, there are partial missing values in grain size, density, and oxygen content. Since the total rare earth content significantly impacts the magnet’s performance, it is also considered a feature variable. The remanence, coercivity, maximum magnetic energy product, and squareness of the sintered NdFeB magnets are used as target variables, while the remaining 29 variables are considered feature variables.

To ensure consistent results for each training session, we divided the data into 70% training set and 30% test set, and set a random seed. We constructed a multi-head attention neural network regression model and trained traditional machine learning model(XGBoost) and a deep neural network regression model for comparison. The XGBoost model uses a greedy algorithm^27^ to calculate the optimal split point of a feature that maximizes the decrease in the objective function^28^. The split function is non-differentiable, and during the split, the model automatically assigns all missing values in the dataset to the same leaf node. Therefore, there is no need to handle missing data or perform normalization, but a regularization term^29^ must be added to prevent overfitting. On the other hand, the neural network regression model calculates the loss function through a gradient descent algorithm^30^, which requires handling missing values and normalizing the data to ensure consistent gradient update directions and to accelerate model convergence.

### Multi-head self-attention mechanism regression model

**Fig. 8:**
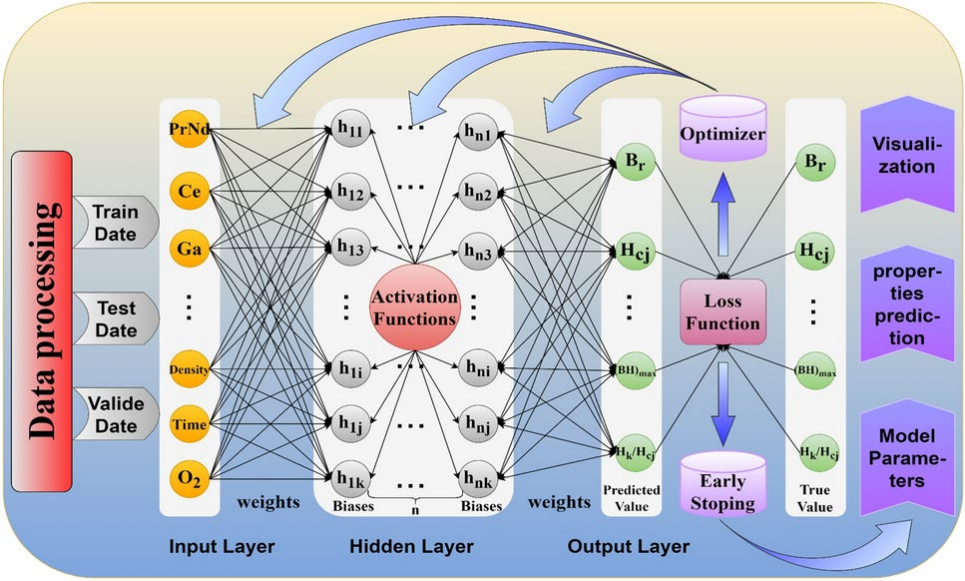
The structure diagram of a full connected neural network regression model.

To construct a multi-head attention neural network regression model, it is necessary to design a full connected neural network regression model first. The architecture of the model as shown in Fig. 8, which consists of modules including data preprocessing, fully connected neural networks^31^, loss functions^32^, optimizers^33^, early stopping mechanisms^34^, and visualization.

The model uses the Xavier^35^ method to initialize the weights and biases in the network based on the dimensions of each layer. It updates the model parameters using the backpropagation algorithm^36^ combined with the Adam optimizer^37^ to minimize the loss function. The early stopping mechanism is triggered to halt the training of the model when the loss no longer decreases over several consecutive iterations, resulting in the final predictive model.

**Fig. 9:**
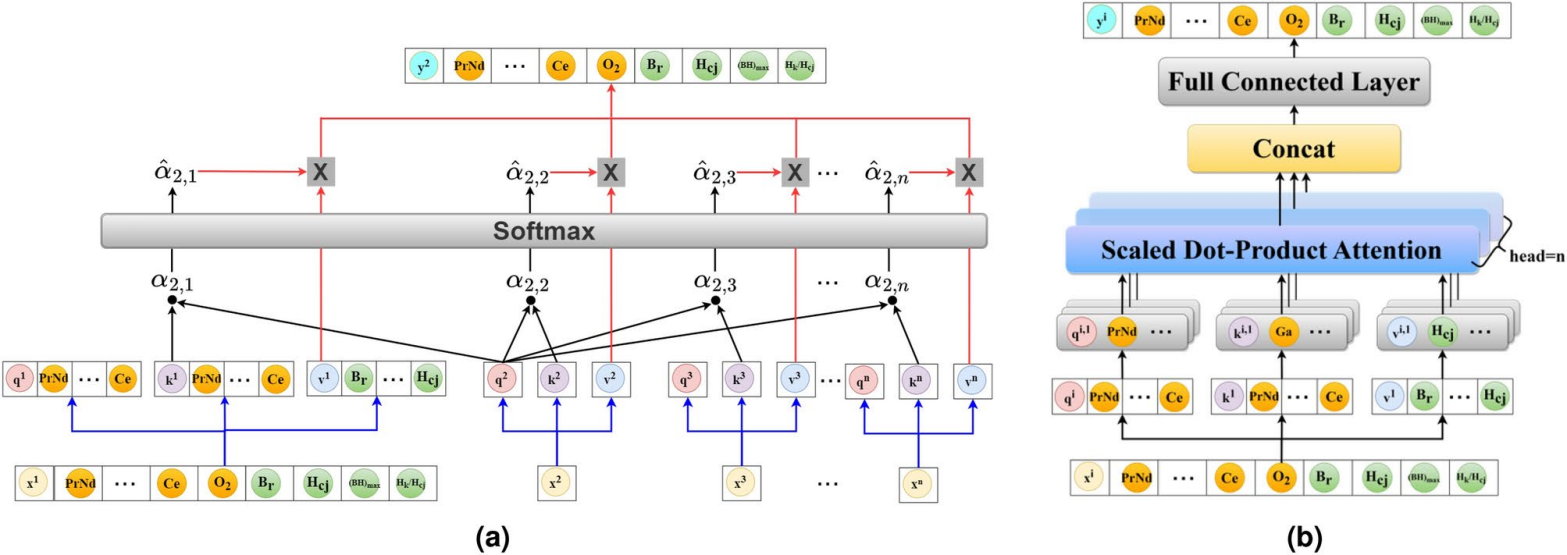
Figure (**a**) represents the schematic diagram of the scaled dot-product attention model, and figure (**b**) shows the structure diagram of the multi-head attention mechanism regression model.

Since the initialized weight parameters are random, the functioning mechanism of the fully connected network layers is difficult to understand. By using the attention mechanism to transform the dependencies between features into attention weights, replacing the randomly initialized weights in the neural network, the interpretability of the model can be improved to some extent. The attention weights are calculated using the scaled dot-product attention model^38^ as shown in Eq. (1).

1 $$\begin{aligned} \text {Attention}(Q, K, V) = \text {softmax}\left( \frac{QK^T}{\sqrt{d_k}}\right) V \end{aligned}$$

where Q, K, and V represent the query, key, and value vectors, respectively, and $d_k$ represents the dimension of the key vector. To understand the mechanism of the model more intuitively, Fig. 9 illustrates the schematic diagram of the scaled dot-product attention mechanism and the structure of the multi-head attention^39^regression model.

Figure 9a shows how the scaled dot-product attention mechanism was used to predict the target value of the second sample. It is done by performing a dot product operation between the query vector of this sample and the key vectors of all other samples to obtain attention scores. The softmax function was then used to convert the attention scores into a weight matrix. Finally, the attention weights were utilized to perform a weighted sum of all value vectors to predict the target value. The figure indicate that Q and K of each sample are equal and set as feature vectors, while V was set as the target vector.

To better capture the nonlinear relationships between features, Q, K, and V were divided into multiple smaller feature subspaces, with each space referred to as a head, establishing the multi-head attention regression model as shown in Fig. 9b. Each head in the model has independent query vector, key vector, and value vector. The attention scores of each head was calculated using the scaled dot-product attention mechanism, and all head share the weight parameters, enabling parallel data processing and accelerating model convergence. Finally, the mapping relationships of all heads are concatenated to form the attention weight matrix. as shown in Eq. (2)

2 $$\begin{aligned} \text {MultiHead}(Q, K, V) = \text {Concat}(\text {head}_1, \text {head}_2, \ldots , \text {head}_h) W_O \end{aligned}$$

where $\text {head}_i$ represent the output of the i-th attention head and $W_O$ represent output weight matrix. The gradient descent algorithm of the fully connected neural network was used to update the weight parameters of the Q, K, and V linear layers and the attention weight parameters, resulting in an optimal parameter model for prediction with the loss function as shown in Eq. (3).

3 $$\begin{aligned} \text {Loss} = \frac{1}{N} \sum _{i=1}^{N} (y_i - \hat{y}_i)^2 \end{aligned}$$

where N represent Number of samples in the dataset, $\text {y}_i$ represent actual value for the i-th sample, $\hat{y}_i$ represent the predicted value for the i-th sample and $(y_i - \hat{y}_i)^2$ represent the squared error for the i-th sample.

## Data Availability

The datasets used and/or analyzed during the current study are available from the corresponding author on reasonable request.
