# Author Correction: Performance prediction of sintered NdFeB magnet using multi-head attention regression models

**DOI:** 10.1038/s41598-024-83660-5

**Published:** 2024-12-20

**Authors:** Qichao Liang, Qiang Ma, Hao Wu, Rongshun Lai, Yangyang Zhang, Ping Liu, Tao Qi

**Affiliations:** 1 Department of Rare Earth, Jiangxi University of Science and Technology, Ganzhou, 341000 China; 2 Department of Physics, Ganjiang Innovation Academy, Chinese Academy of Sciences, Ganzhou, 341119 China; 3 Department of Technology, Jiangxi Guanying Intelligent Technology Co.Ltd., Ganzhou, 341000 China

Correction to: *Scientific Reports* 10.1038/s41598-024-79435-7, published online 21 November 2024

In the original version of this Article, Qiang Ma and Tao Qi were omitted as corresponding authors. Correspondence and requests for materials should also be addressed to maqiang@gia.cas.cn and tqi@gia.cas.cn.

The original Article has been corrected.

